# Role of Environmental Pollutants in Liver Physiology: Special References to Peoples Living in the Oil Drilling Sites of Assam

**DOI:** 10.1371/journal.pone.0123370

**Published:** 2015-04-13

**Authors:** Tapan Dey, Kabita Gogoi, Balagopalan Unni, Moonmee Bharadwaz, Munmi Kalita, Dibyajyoti Ozah, Manoj Kalita, Jatin Kalita, Pranab Kumar Baruah, Thaneswar Bora

**Affiliations:** Biotechnology Division, Council of Scientific & Industrial Research—North-East Institute of Science and Technology, Jorhat, Assam, India; University of Louisville, UNITED STATES

## Abstract

The populations residing near polluted sites are more prone to various types of diseases. The important causes of air pollution are the suspended particulate matter, respirable suspended particulate matter, sulfur dioxide and nitrogen dioxide. As limited information is available enumerating the effect of these pollutants on liver physiology of the population living near the polluted sites; in the present study, we tried to investigate their effect on liver of the population residing near the oil drilling sites since birth. In this study, a randomly selected 105 subjects (46 subjects from oil drilling site and 61 subjects from control site) aged above 30 years were taken under consideration. The particulate matter as well as the gaseous pollutants, sulfur dioxide and nitrogen dioxide, were analyzed through a respirable dust sampler. The level of alkaline phosphatase, alanine transaminase and aspartate transaminase enzymes in serum were measured by spectrophotometer. The generalized regression model studies suggests a higher concentration of respirable suspended particulate matter, suspended particulate matter and nitrogen dioxide lowers the alkaline phosphatase level (p<0.0001) by 3.5 times (95% CI 3.1-3.9), 1.5 times (95% CI 1.4 - 1.6) and 12 times (95% CI 10.74 -13.804), respectively in the exposed group. The higher concentration of respirable suspended particulate matter and nitrogen dioxide in air was associated with increase in alanine transaminase level (p<0.0001) by 0.8 times (95% CI 0.589-1.049) and by 2.8 times (95% CI 2.067-3.681) respectively in the exposed group. The increase in nitrogen dioxide level was also associated with increase in aspartate transaminase level (p<0.0001) by 2.5 times (95% CI 1.862 – 3.313) in the exposed group as compared to control group. Thus, the study reveals that long-term exposure to the environmental pollutants may lead to liver abnormality or injury of populations living in polluted sites.

## Introduction

Oil Spillage has always been a prominent reason for causing air pollution around the oil drilling sites. Many epidemiological studies have suggested the environmental pollutants due to oil spillage play a major role in developing various kinds of respiratory problems such as chronic obstructive pulmonary disease (COPD), asthma and other related diseases [1]. Many investigators have correlated the concentration of environmental pollutants to different respiratory diseases of human beings [2, 3]. Recently, few epidemiological studies showed the impact of large marine oil spills affecting the health of cleanup operators [1, 4]. They studied the persistence of functional and biological respiratory health effects after 6 years of completion of the cleanup work and found no clear differences between highly exposed and moderately exposed cleanup workers [4]. It is also reported that, the higher concentrations of sulphur dioxide (SO_2_) and nitrogen dioxide (NO_2_) pollutants were significantly related to symptoms of respiratory ailments in children [5].

Among other investigations, Amdur et al. (1953) [6]examined the responses of breathing exposed up to 8 ppm SO_2_ in one of the first controlled studies in male populations. They observed that SO_2_ is responsible for a change in respiratory pattern of the subjects. Schwartz et al. (1987) [7]examined daily mortality and suspended particulate matter level in air using Poisson regression model in Philadelphia and have found a significant association of suspended matter level and mortality. Extensive human exposures to mixtures of ozone and NO_2_ have typically resulted in declined pulmonary function [8, 9]. However, the data for human exposures to NO_2_ lacks consistency and reproducibility as the two groups initially reported small changes in lung functions associated with NO_2_ exposure in asthmatics [9, 10] but subsequently they were unable to replicate their findings. Nevertheless, NO_2_ exposure can make the subjects more susceptible to asthma and COPD [11].

Gene susceptibility along with environmental contaminants was found to be responsible for the severity of COPD [12, 13]. Although, there is significant data suggesting the effect of environmental pollutants in respiratory diseases, the data on the impact on the liver at blood biochemical level is limited. Earlier, we conducted a number of surveys around different polluted sites (oil drilling site, coal mines, paper mill area) where we recorded a large number of the population facing serious health related problems due to environmental pollutants. During the health survey, it was observed that most of the populations residing near the oil drilling sites have the history of anorexia, nausea, progressive weakness and weight loss. The manifestations observed in the exposed group were mainly due to hepatobiliary insufficiency. Therefore, the present study was taken up to assess long-term exposure effect of environmental pollutants on liver parameters among the population residing near the oil drilling sites since birth.

## Methods

### Health survey and sample collection

A health survey was conducted among the inhabitant residing near the oil drilling sites at Borholla of Assam. People aged more than 30 years were considered for the study. The study site is within 5 Sq. Km from the drilling site. 105 subjects were enrolled in the study (46 samples from near oil drilling site and 61 subjects from control site). The subjects are selected independently with equal probabilities applying Simple Random Sampling (SRS). The control site was 120 Kilometers away from the oil drilling sites. There was no existence of any industries near the residence of control population.

Collection of blood sample was done according to the guidelines of Indian Council of Medical Research and with the approval of the Institutional Ethics Committee, CSIR-North East Institute of Science & Technology, Jorhat. Written consents were taken from the blood donors for the study.

### Environmental pollution assessment

Environmental pollutants were assessed with the help of a Respirable Dust Sampler (Envirotech, Model APM 460 BL) and data was calculated for respirable suspended particulate matter (RSPM) and suspended particulate matter (SPM). Determination of SO_2_ in ambient air was done with the help of modified West and Gaeke Method and NO_2_ were determined by Sodium Arsenite Method (as per manufacturer’s instructions by the Central Pollution Control Board, Government of India). A distant area located at 120 Kilometers away from the polluted site served as control site for air analysis. The air dust samples were collected in glass microfiber filter papers of pore size 1.6 μM.

### Blood Biochemistry assessment

The liver function test parameters considered for study were alanine transaminase (ALT), aspartate transaminase (AST), alkaline phosphatase (ALP), total bilirubin (TB), direct bilirubin (DB), total protein (TP) and albumin (ALB). Biochemical analysis was done for all the blood samples collected from the subjects at polluted sites as well as the control sites.

Modified IFCC method (UV Kinetic) was used to find out ALT and AST level in blood serum at 340 nm. The Modified Kind & King's method (End Point Abs.) was used to find out alkaline phosphates in serum at 510 nm. Modified Jendrassik & Grof's method (End Point) was used to find out DB & the TB level in serum at 546 nm. The Bromocresol Green (BCG) method (End Point) was used to find out ALB levels in serum at 630 nm. The TP level determination was carried out through Biuret method (End Point) and measured at 550 nm. The reference ranges for all the parameters were set according to Longo et al. (2011) [14] as Total Bilirubin (TB): 0.4 – 1mg/dl, Direct Bilirubin (DB): 0.0–0.2 mg/dl, Total Protein (TP): 6.0–8.0 gm/dl, Albumin 3.5–5.2 gm/dl, ALT: 37U/L, AST: 37U/L and ALP: 100–280 U/L. All above assays were taken by using a Spectrophotometer (PG Instruments Ltd.).

### Statistical analysis

The association of ALP, ALT, AST, TB, DB, TP and ALB with the exposed/unexposed population was determined by Student’s t-test. The generalized regression analysis (95% Confidence Interval and p value) were calculated to find out the association of the liver parameters with the levels of respirable dust particulate matter, suspended particulate matter and NO_2_. The statistical analyses were done by using SPSS v14 and GraphPad—Prism 5 software.

## Results

The baseline characteristics of both the exposed and control groups were shown in Table 1. The body mass index (BMI) of the exposed group was found to be significantly lower (P value < 0.05) as compared to control population irrespective of gender. The mean age difference between the exposed and control population was statistically insignificant.

**Table 1:** Baseline Characteristics of exposed and unexposed group.

	Exposed Group	Unexposed Group(n = 61)	P value
	(n = 46)		
Male	48.89±1.81 (n = 26)	48.21±1.75 (n = 29)	P = 0.79^NS^
Female	41.80±1.93 (n = 20)	46.72±2.06 (n = 32)	P = 0.11^NS^
Mean Age	48.89 (30–73)	48.21 (30–80)	
Body Mass Index (BMI)			
Male: Male	15.37±0.78	19.44±1.01	P = 0.0029**
Female: Female	14.70±0.76	18.26±0.91	P = 0.0087**

All values are expressed as Mean ± SEM (Standard Error of Mean), P value <0.05 considered as significant;

** = Significant,

^NS^ = Not Significant, n = number of samples/observation (Values were expressed up to two decimal point).

Air sample analysis was done throughout a year both in oil drilling sites and in the control sites for all the variables such as respirable suspended particulate matter, suspended particulate matter, SO_2_ and NO_2_. Air samples collected from 120 Kilometers distant areas was served as control. The concentrations of all the variables under consideration were found to be higher than the control site. The level of respirable suspended particulate matter and suspended particulate matter was found to be higher two times and three times, respectively in drilling site than the control site (Table 2). The concentrations of the gaseous compound, NO_2,_ were found to be two times higher than the control site.

**Table 2:** Comparison of air components concentration present in polluted area (oil drilling sites) and control site.

Sites	RSPM (μg/m^3^)	SPM (μg/m^3^)	NO_2_ (μg/m^3^)	SO_2_ (μg/m^3^)
Unexposed area	29.10±0.50	41.40±1.20	14.40±0.20	0.02±0.01
Exposed area	69.80±0.40**	132.50±0.90**	26±0.70**	0.86±0.04**

All values are expressed as Mean ± SEM (Standard Error of Mean),

** = Statistically significant with P <0.0001) (Values were expressed up to two decimal point).

The blood biochemical level studies showed that the mean values of ALT (59.30±4.90) and AST (61.02±3.90) are higher and ALP (48.04±7.175) level is lower in exposed population than the control population. Student t-test revealed that these differences were statistically significant (Table 3). Other features such as bilirubin (direct and total), albumin and total protein levels of the exposed population were found to be in normal range and lie statistically different from controls.

**Table 3:** Comparison of biochemical tests of the entire seven variables among exposed and unexposed group by Student’s t-test

	ALP	ALT	AST	TB	DB	TP	ALB
Unexposed	190.40±5.59	25.96±1.79	31.00±2.22	1.04±0.13	0.28±0.04	6.62±0.08	4.07±0.04
Exposed	48.04±7.18**	59.30±4.90**	61.02±3.90**	0.81±0.05^NS^	0.27±0.02^NS^	6.80±0.17^NS^	3.08±0.08^NS^

All values are expressed as Mean ± SEM (Standard Error of Mean),

** = Highly significant with P <0.0001,

^NS^ = Not Significant (Values were expressed up to two decimal point).

The effect of the environmental pollutants on the ALP, AST and ALT was also evaluated by regression analysis (Table 4). The results of the generalized regression analysis reveals that environmental RSPM, SPM and NO_2_ levels were significantly linked to up regulation and down regulation of ALP, ALT and AST levels in exposed population.

**Table 4:** Associative studies of alkaline phosphatase (ALP), alanine transaminase (ALT) and aspartate transaminase (AST) with the environmental variables by generalized regression analysis.

Enzymes	Variables	Values of estimated Coefficient (β)	95% Confidence Interval (CI)
ALP	RSPM	-3.498**	-3.934 to -3.062
	SPM	-1.563**	-1.578 to -1.368
	NO_2_	-12.273**	-13.804 to -10.74
ALT	RSPM	0.819**	0.589 to 1.049
	SPM	0.366**	0.263 to 0.469
	NO_2_	2.874**	2.067 to 3.681
AST	RSPM	0.738**	0.531 to 0.944
	SPM	0.329**	0.237 to 0.422
	NO_2_	2.588**	1.862 to 3.313

**Statistically significant P< 0.001.

Negative values indicates decrease in level.

## Discussion

The serum ALT and ALP levels were considered to be specific for hepatocellular injury with a few exceptions [15]. However, the degree of enzyme levels classified into mild, moderate or markedly high levels can be helpful to distinguish between different causes of liver diseases [16]. Elevations of the serum AST were reported in viral hepatitis as well as other liver diseases [17, 18]. AST and ALT are the hepatic enzymes that catalyse the transfer of amino groups to form the hepatic metabolites pyruvate and oxaloacetate, respectively. Both the ALT and AST are released from damaged hepatocytes into the blood after hepatocellular injury or death leading to their higher concentration in blood [19]. Till now, most of the investigations were done to study the effect of environmental pollutants on lung diseases. Thus, in this study we have evaluated the impact of environmental pollutants such as NO_2_, RSPM and SPM present in air surrounding the oil drilling sites on liver abnormality or injury of the population living near those sites. Here, we have found that the presence of air pollutants in the environment does play an important role in liver abnormality or injury to the inhabitant as compared to control groups residing in non-polluted area.

The investigation carried out by Bai et al. (2004) [20] through haematoxylin eosin (HE) staining and transmission electron microscopy (TEM) analysis found that SO_2_ inhalation can cause liver injury. Likewise, exposure to NO_2_ also affects the microsomal electron-transport systems in the liver [21].

Different epidemiological studies reported the fine particles (10μM) are associated with most health problems, such as heart and lung diseases [22–24], diabetes [25], premature birth and low birth weight [26], cancer [27] and non-alcoholic fatty liver disease [28]. Much like particulate matters, ambient SO_2_ concentration was associated with mortality [29], lung cancer [30], respiratory diseases [31, 32] and stillbirth [33]. Further, the ambient NO_2_ concentrations were associated with pulmonary defects [34, 35], cardiovascular complications [36], mortality [37] and offspring’s birth weight [38].

**Fig 1:**
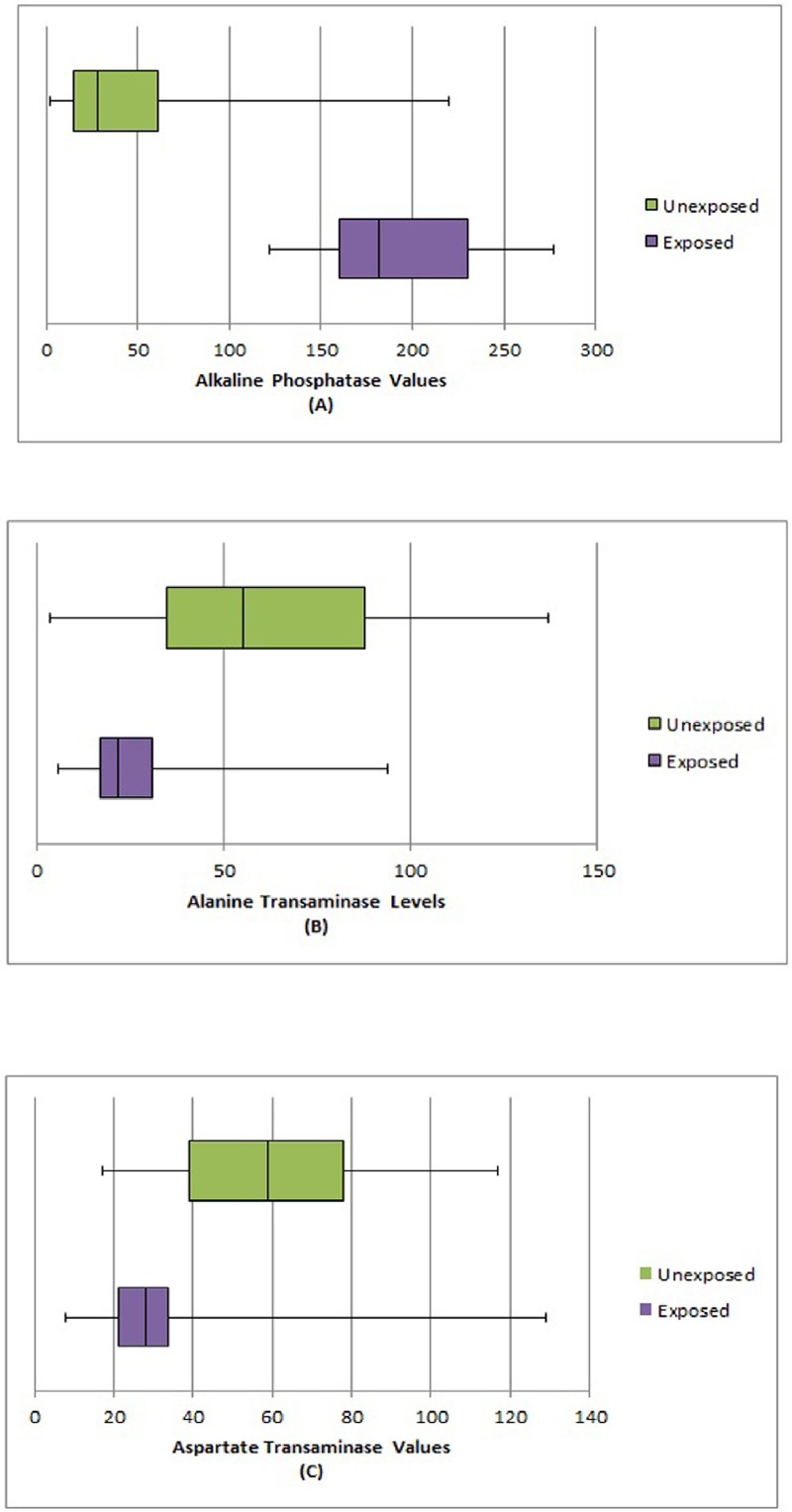
(A, B, C) Box and Whisker plot of different liver enzymes level in unexposed and exposed population.

Our earlier studies on the same oil drilling site, we have recorded a higher level of iron, nickel, potassium, magnesium, calcium, manganese and selenium present in the soil samples as compared to control site. We also found a higher concentration of lead, arsenic and manganese in water samples collected from the same area [39]. Manganese, lead, mercury are found to have most vital hepatotoxic effect associated with up and down regulation of liver enzymes [40, 41]. Based on the biochemical analysis we found low-level of nutritional parameters such as protein, carbohydrates and lipids in the vegetables. In this study, we targeted the population of age group more than 30 years, so as to assess the long-term exposure effect of these pollutants on liver of that exposed group. The BMI of the exposed group when compared with the control group, it was recorded that the exposed population group are underweight (Table 1). We found the mean values of ALT (P< 0.0001) and AST (P< 0.0001) was higher whereas the mean value of ALP (P< 0.0001) was lower in exposed population as compared to control population. The box and whisker plots were shown for all three enzymes in Fig 1A, 1B and 1C.

Since the concentration of RSPM, SPM, SO_2_, NO_2_ were higher in the polluted area as compared to control area, we further evaluated the effect of these pollutants on ALP, AST and ALT level (taking the Student t-test’s results as the basis) by generalized regression analysis. The generalized regression analysis between the pollutants and the liver parameters showed an increase in the RSPM level which led to significant decrease of 3.5 times ALP level (95% CI 3.1–3.9) in the population living near the polluted site. Similarly, the SPM level was found to be associated with the decrease of 1.5 times ALP level (95% CI 1.4–1.8) in exposed population as compared to control population (Table 4). The NO_2_ level was associated with a 12 times decrease in the ALP level (95% CI 10.74 to 13.804) in the exposed population (Table 4). Whereas, the increase of RSPM level was associated with increased blood ALT level by 0.8 times (95% CI 1.049 to 0.589) and an increase of the NO_2_ level (Table 2) increased the ALT level by 2.8 times (95% CI 2.067 to 3.681) (Table 4). The increase in NO_2_ level (Table 2) was also associated with increased AST level by 2.5 times (95% CI 1.862–3.313).

As our study was an epidemiological approach there were certain limitations. Liver biopsy of the consent populations having abnormal liver enzymes was not possible for us. We did not have any emphasis on the gender and occupation of the exposed population and only considered their long-term exposure as they have stayed in that polluted area since birth. Further studies based on clinical approach of the present findings on pathways relating gaseous pollutants and liver enzymes will be useful.

## Conclusion

The long term exposure of particulate matter and gaseous pollutants may play an important role in liver physiology of the inhabitant population living near the polluted sites.
